# Patient Education Materials for Immobilisation Masks in Radiation Therapy for Adult Head and Neck Cancer Patients: A Scoping Review

**DOI:** 10.1007/s13187-024-02436-7

**Published:** 2024-04-09

**Authors:** Lucy Wood, Ruby Holman, Uyen Nguyen, Helen Nguyen, Aurora Senaratna, Misha Adams, Apajok Apath

**Affiliations:** Allied Health and Human Performance, University of South Australia, Adelaide, South Australia Australia

**Keywords:** Immobilisation Masks, Head and Neck Cancer, Patient Education Materials, Radiation Therapy, Patient Anxiety

## Abstract

Immobilisation masks (IMs) are used for people with head and neck cancer (HNC) undergoing radiation therapy (RT) treatment to ensure accuracy and reproducibility between treatments. Claustrophobia-related mask anxiety in HNC patients is common and can compromise treatment due to patient distress. This scoping review aimed to describe the content of publicly available Patient Education Materials (PEMs) for people with HNC undergoing RT. Three search engines (Bing, Yahoo, and Google) were systematically searched using standard terms. PEMs in audio-visual or written formats were eligible for inclusion if the target readership was adults with HNC and included content on IMs for RT. Content was appraised using the Patient Education Materials Assessment Tool for Printable and Audio-Visual Materials to assess understandability and actionability. In total, 304 PEMs were identified of which 20 met the inclusion criteria. Sixteen PEMs were webpages, three were PDF format, and one was a standalone video. The understandability and actionability of PEMs ranged between 47 to 100% and 0 to 80%, respectively. PEMs authored by Foundations/Organisations scored higher in understandability (80–100%) and were more likely to discuss mask anxiety coping strategies. In comparison, News sites and IM manufacturers published PEMs with the lowest understandability scores (20–80%). The significant variations in the quality of IM PEMs identified suggest that some sources may be more effective at informing patients about IMs. Although multiple aspects of the PEMs were consistent across the reviewed materials, many PEMs lacked information, and a stronger focus on understandability and actionability is required.

## Introduction

Head and neck cancer (HNC) is an umbrella title given to cancers of the sinuses, pharynx, larynx, mouth, and salivary glands [1]. Recent studies indicate that rates of HNC are increasing, with a current global yearly incidence of around 660,000 new cases, ranking HNC as the seventh most common cancer [1]. HNC often utilises multiple approaches for treatment, with surgery, radiation therapy (RT), and systemic therapy commonly used [2]. RT has an integral role in HNC treatment and, considering tumour characteristics, can be implemented preoperatively, adjuvant to other treatment methods or be the primary treatment. RT provides a highly conformal radiation dose to the treatment area whilst sparing surrounding healthy tissue and critical organs. With typical target volume expansions of < 5 mm used in the treatment plan, positioning of the patient and immobilisation of the treatment area are crucial [3]. The use of an immobilisation mask (IM) is considered standard practice and essential to accurately reproduce the position of the patient in each treatment and to avoid irradiating critical structures [4]. Patient-specific thermoplastic mesh masks, which cover the entire face, and often the neck and shoulders, are created during the initial computed tomography (CT) simulation of a patient’s RT journey [4]. During treatment sessions, masks are temporarily secured to the treatment couch, with the patient positioned inside [4]. Closed IMs cover the whole head or head and shoulders with the thermoplastic, whilst open IMs leave the face uncovered. It has been suggested open IMs may offer improved comfort and decreased levels of anxiety [3].

The current literature sheds light on the significance of claustrophobia-related mask anxiety in people living with HNC undergoing RT in conjunction with potential coping strategies. It has been shown in numerous studies that patients consistently report emotional distress, claustrophobia, anxiety, and vulnerability because of IMs [1, 5–11]. Physical symptoms such as sweating, an elevated heart rate, and abnormally rapid rates of breathing are also commonplace [5]. Ramifications of these manifestations include patient distress, costly treatment disruptions, increased patient movement, and non-compliance to the treatment plan potentially resulting in poorer outcomes [1].

Studies have estimated that “mask anxiety” can be seen in 14 to 58% of people required to use IMs during RT, noting that a diagnosis of HNC and the reality of undergoing RT already brings psychological challenges for patients [4, 5]. Although there are strategies to manage mask anxiety between centres, people continue to report high levels of feelings of unpreparedness [1]. Additionally, the management of psychological distress through the provision of pre-treatment education and continual support throughout treatment has been widely acknowledged and accentuated in the literature [8]. Klug et al. proposed that PEMs are an area of improvement that has great potential to effectively increase preparedness and lower mask anxiety in HNC patients [1].

In the Australian and New Zealand context, Keast et al. and Forbes et al. suggested an association between patient anxiety and inconsistency in the information given in patient education materials (PEMs) on IMs [4, 12]. Keast et al. accentuated the significance of resolving mask anxiety to lessen the persistent impact of traumatic experiences in HNC patients, post-treatment [12]. Attempts to provide patients with more information regarding IMs have been suggested to be beneficial in improving patient’s treatment-related decision-making and minimising treatment interruptions [4]. Yet, there is a scarcity of current or in-progress scoping reviews or systematic reviews on the quality of PEMs published on the topic of IMs. As such, this review aims to describe PEMs on the topic of IMs publicly available to people living with HNC undergoing RT. The review question guiding this review is “How do PEMs on the topic of IMs available to HNC patients undergoing radiation therapy vary?”.

## Methods

A preliminary search of PROSPERO, MEDLINE, the Cochrane Database of Systematic Reviews, and JBI Evidence Synthesis did not identify any protocols specific to this review question. This scoping review was conducted in line with the PRISMA scoping review guidelines [13]. A protocol for this scoping review detailing the objectives, inclusion criteria, and methods was registered in advance on the open science framework (Registration DOI: https://doi.org/10.17605/OSF.IO/GAUXE).

### Eligibility Criteria

PEMs were eligible for inclusion within this scoping review if they included content focussed upon the use of open and/or closed IMs for adults (> 18 years of age) with HNC and undergoing RT, written in English, publicly available at no cost, and published/revised from 1 January 2013 to 1 January 2024 to ensure currency of information. PEMs in both printable format (such as infographic, downloadable PDF or downloadable webpages) and audio-visual format (such as podcast or video) were eligible. PEMs focussed upon the use of paediatric IMs or not specific to RT were not eligible for inclusion in this review.

### Information Sources

Sources were limited to grey literature authored by credible organisations. PEMs were sourced from search engines including Google, Bing, and Yahoo, which ensured all sources were accessible to the public.

### Search Strategy

Development of the search strategy included both preliminary searches of common public-facing search engines (Google, Yahoo, and Bing) and consultation with an academic librarian at the University of South Australia. The population, concept and context framework was used for this review where population (adults with HNC undergoing radiation therapy), concept (patient education materials), and context (IMs used in radiation therapy) helped form the search strategy and search terms.

Three different search statements containing alternative keywords relating to the research question were searched on Google, Bing, and Yahoo. The same statements were searched by one author per search engine (UN for Google, HN for Yahoo, AS for Bing), with no advanced settings applied. The statements “patient educational material on immobilisation facemask head and neck cancer”; “factsheet on radiation therapy mask HNC” and “patient resource radiation mask melanoma of head and neck cancer” were used for the search strategy.

Search engine results were sorted from most to least relevant; Pham et al. recommended limited search results to the first 40 on the basis that relevance will plummet past the first five pages [14]. The first 40 results from each search statement were stored on a shared Google document.

### Study Records

Two authors were assigned to the results of each search engine and independently reviewed each result against the eligibility criteria (UN and RH assigned to Google, HN and MA assigned to Yahoo, and AA and AS assigned to Bing). Duplicates were manually identified and removed before screening. A third author (AS) was involved in resolving conflicts. PEMs meeting eligibility requirements were evenly divided among authors for data extraction; data was extracted manually and independently (RH and UN assigned to Google, HN and MA assigned to Yahoo, AA and AS assigned to Bing) using a data extraction template, created by RH and UN. Discrepancies were resolved by a third author (MA).

### Data Items

Data was extracted for three domains. All publication dates were present and there was no need to contact authors.

Domain one: publication demographic [title, author(s)/authoring body (categorised as Treatment Provider; Government Body; Foundation/Organisation; Other), year of publication or last update, country of origin].

Domain two: format [word count, number and type of figures/visual aids (AV; schematic; real-life demonstrations)].

Domain three: content [IM fitting; the purpose of IM; RT background; patient experiences; IM-related patient preparation; RT-related patient preparation; mask anxiety/claustrophobia; IM-related coping mechanisms; RT-related coping mechanisms, number and type of links to helpful resources (helplines; treatment information; further PEMs; articles), and type of IM discussed (open; closed; both)].

### Assessment of Information Quality

An appraisal of the information quality of the PEMs eligible for inclusion was undertaken using the “Patient Education Materials Assessment Tool for Printable Materials” (PEMAT-P) and “Patient Educational Materials Assessment Tool for Audio-visual Materials” (PEMAT-A/V) [15]. PEMAT-P and PEMAT-A/V assessed PEMs on understandability and actionability. Each PEM was reviewed and appraised by two authors, each acting independently. On completion, authors met to discuss and compare results with discrepancies resolved by a third author assigned to independently appraise the PEM.

The definitions of understandability and actionability have been adapted from Shoemaker, Wolf, and Brach and are stated below [16].

Understandability: PEMs are *understandable* when consumers of diverse backgrounds and various levels of health literacy can process and explain key messages.

Actionability: PEMs are *actionable* when consumers of diverse backgrounds and varying levels of health literacy can recognise what they can do based on the information presented to them.

PEMAT-A/V measures understandability using 19 items, and actionability using 4 items [15]. PEMAT-P measures understandability using 19 items, and actionability using 7 items [15]. Each PEM is scored numerically per item to provide a percentage score of understandability and actionability. High scores indicate high understandability and actionability for both PEMAT-P and PEMAT-A/V. The potential final score range was 0 to 100%.

The process for appraising each PEM using the PEMAT-P and PEMAT-A/V required each assessor to (1) read/view the PEM, (2) assign the PEM to either the PEMAT-P or PEMAT-A/V, (3) score each PEM item for specific PEMAT criterion for both understandability and actionability (Disagree =  − 1 red; Agree =  + 1 green; Not Applicable = 0 yellow), and (4) calculate the PEMs understandability and actionability scores.

The Cochrane traffic-light system was adapted to present the results from each item of the PEMAT tools.

### Data Synthesis

Results were reported in tabulation, graphs, and data maps followed by narrative descriptions. A PRISMA flowchart presented the flow of information through the review. The characteristics, understandability and actionability scores, and content of the PEMs were tabulated. Narrative synthesis of the characteristics and variabilities in content and value of the PEMs was conducted. Understandability and actionability were compared between authoring bodies and country of origin. Authoring bodies were further compared in terms of their content discussed, visual aids, and links provided. Additional focus was placed on reporting of mask-anxiety and associated coping mechanisms, which were further synthesised graphically.

### Amendments to the Protocol

Alteration of the eligibility criteria permitted the inclusion of PEMs published from 2013 onward (previously being 2015). The inclusion criteria were altered to allow the inclusion of PEMs that had a strong focus on IMs for HNC RT, as opposed to being solely focused on IMs.

The PEMAT-P and PEMAT-A/V appraisal process used a Cochrane traffic-light system adaption instead of a table, as this permits a visual representation of the appraisal process.

## Results

### PEM Selection

**Fig. 1:**
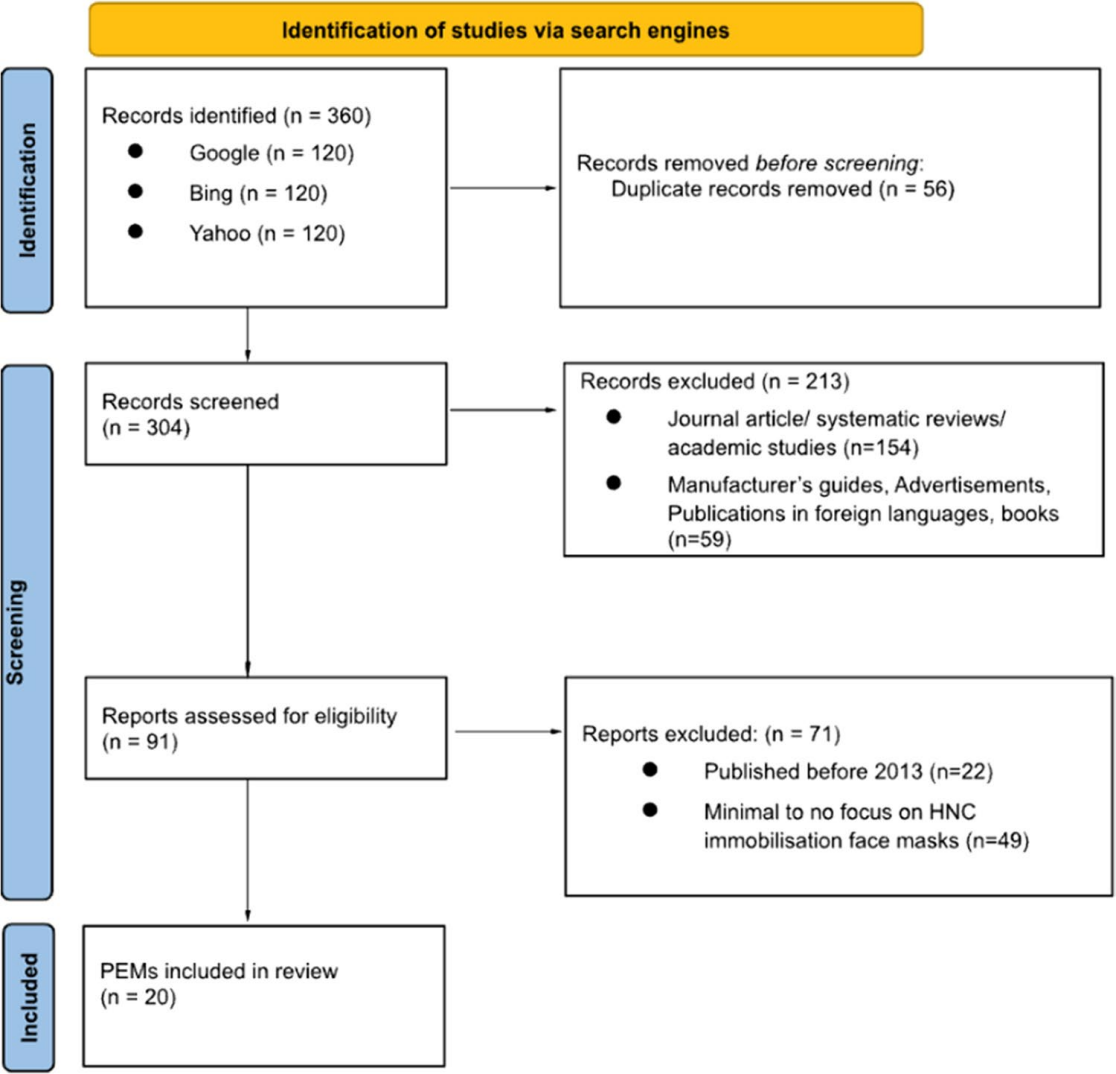
Selection of patient education materials (PEMs) for inclusion in the review in the format of a PRISMA diagram

Figure 1 presents a summary of the flow of information through the search and screening process. Of the 360 original results retrieved from the search, 56 duplicate references were removed before initial screening. Of the 304 records screened, 213 were excluded for various reasons (Fig. 1) and 91 provisionally met eligibility and were reviewed as full text where a further 71 references were excluded (published before 2013 *n* = 22; PEM did not focus upon IM use *n* = 49). Twenty PEMs were left eligible for inclusion in this review.

### Study/Information Type Characteristics

Table 1 presents a summary of PEM demographics. Authoring bodies were categorised: Treatment Providers (*n* = 3), Government Bodies (*n* = 1), Foundations/Organisations (*n* = 9), and Other (universities [*n* = 1], News articles [*n* = 3], mask manufacturing companies [*n* = 3]). Sixteen PEMs (80%) were webpages with 19% (*n* = 3) containing an embedded video or podcast (Table 1). Three PEMs (15%) were a downloadable pdf and 5% (*n* = 1) were a standalone video.

**Table 1:**
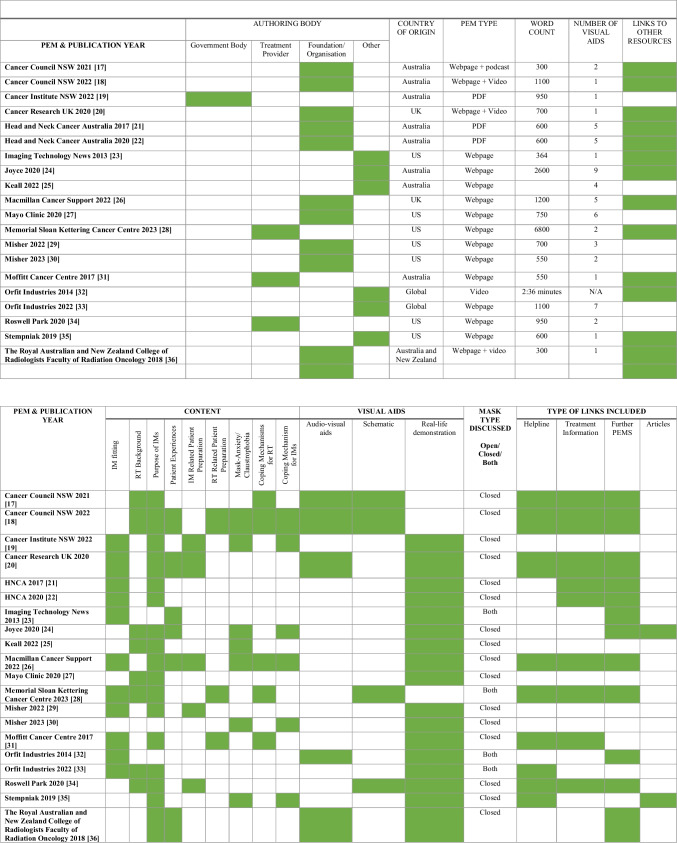
Study characteristics and results

Key: *US*, United States; *UK*, United Kingdom; *NSW*, New South Wales; *RT*, Radiation Therapy; *IM*, Immobilisation Mask; *PEM*, Patient Education Material; HNCA, Head Neck Cancer Australia.

Green shading = yes/applicable

Countries of origin included Australia (*n* = 8), the United States (US) (*n* = 7), the United Kingdom (UK) (*n* = 2), Global (*n* = 2), and Australia and New Zealand collaboration (*n* = 1). PEMs were published between 2013 and 2022. Most PEMs were published in 2022 (*n* = 6) and 2020 (*n* = 5). All 19 (100%) of the printable PEMs had at least one visual aid. The number of visual aids ranged from 1 to 9. PEMs with more than five visual aids were classified as outliers (*n* = 3).

Thirteen (65%) of the included PEMs contained links to other resources. Included PEMs had word counts ranging between approximately 300 and 6800. Seventeen (85%) PEMs were below 1200 words; the remaining two printable PEMs had 2600 and 6800 words.

### Content of PEMs for Immobilisation Masks for People with Head and Neck Cancer Undergoing Radiation Therapy

Seventeen (85%) PEMs contained content on the purpose of IMs, 55% (*n* = 11) on IM fitting, 40% (*n* = 8) on background RT information, 35% (*n* = 7) on mask anxiety/claustrophobia, 30% (*n* = 6) on patient experience, 30% (*n* = 6) on coping mechanisms specific to IMs, 25% (*n* = 5) on coping mechanisms for RT, 25% (*n* = 5) on IM-related patient preparation, and 15% (*n* = 3) for RT patient preparation. The PEMs discussed closed IMs (*n* = 16) or both open and closed IMs (*n* = 4). The sources included either single or multiple links to other resources (*n* = 15), such as helplines (*n* = 9), treatment information (*n* = 9), further PEMs (*n* = 12), and articles (*n* = 2).

Of the nine PEMs authored by Foundations/Organisations, content mainly focused on the purpose of IMs (*n* = 9) and IM fitting (*n* = 7). However, the actionability scores of these PEMs ranged from 20 to 80%. The lowest-scoring PEMs for understandability were those authored by News providers (*n* = 3) with scores ranging from 47 to 73%; these PEMs also scored 0% on actionability.

The purpose of IM use in RT for HNC was the most prevalent content, whereby 85% (*n* = 17) of PEMs discussed the concept. The least commonly discussed category was patient preparation for RT, discussed by only 15% (*n* = 3) of PEMs. PEMs differ significantly in the content discussed, and those authored by Foundations/Organisations were more inclined to explain mask anxiety and suggest coping mechanisms.

### Synthesis of Results/Information Types

Across 20 PEMs with key focuses on IMs for HNC, 35% (*n* = 7) discussed mask anxiety. Only 30% (*n* = 6) suggested coping mechanisms to alleviate mask anxiety. Only one (14%) PEM that discussed mask anxiety was authored by a Government Body, 43% (*n* = 3) were authored by a Foundation/Organisation, and 43% (*n* = 3) were authored by Other that considered this topic. Of the six PEMs that suggested mask anxiety coping mechanisms, 16% (*n* = 1) were authored by a Government Body, 50% (*n* = 3) were authored by a Foundation/Organisation, and 33% (*n* = 2) were authored by Other. No PEMs authored by treatment providers discussed mask anxiety or suggested coping mechanisms.

### Appraisal of Information Quality

**Fig. 2:**
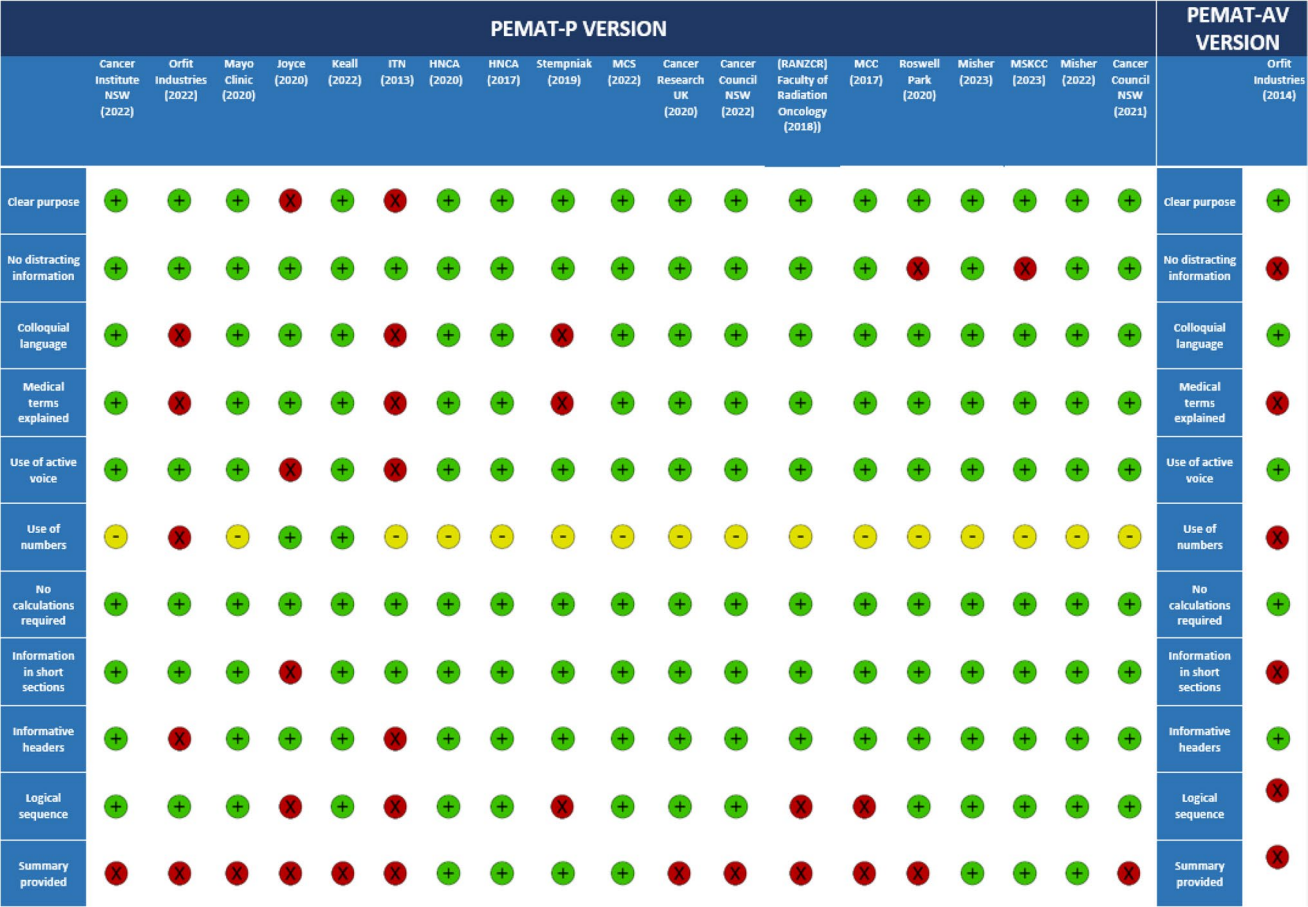

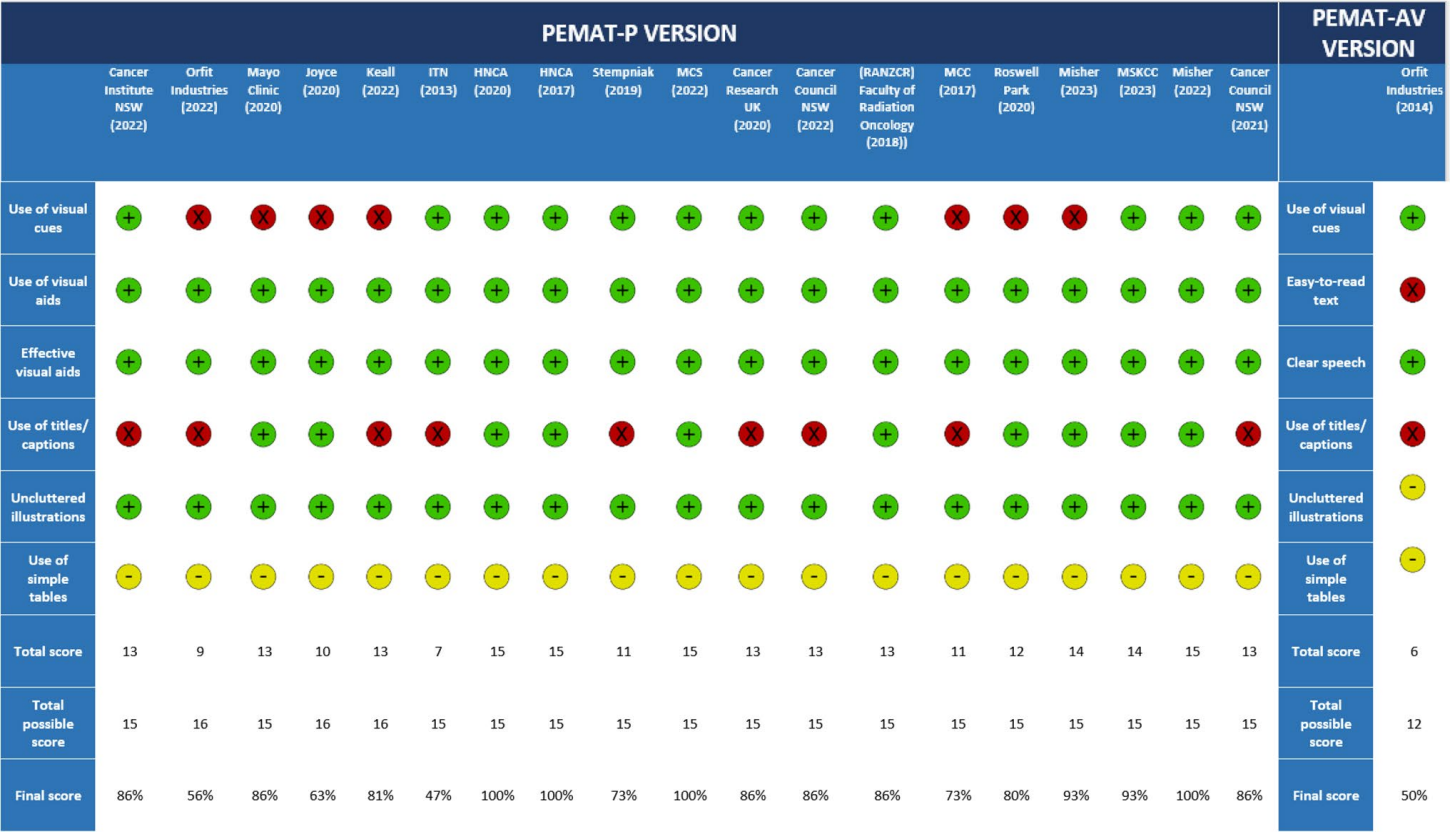

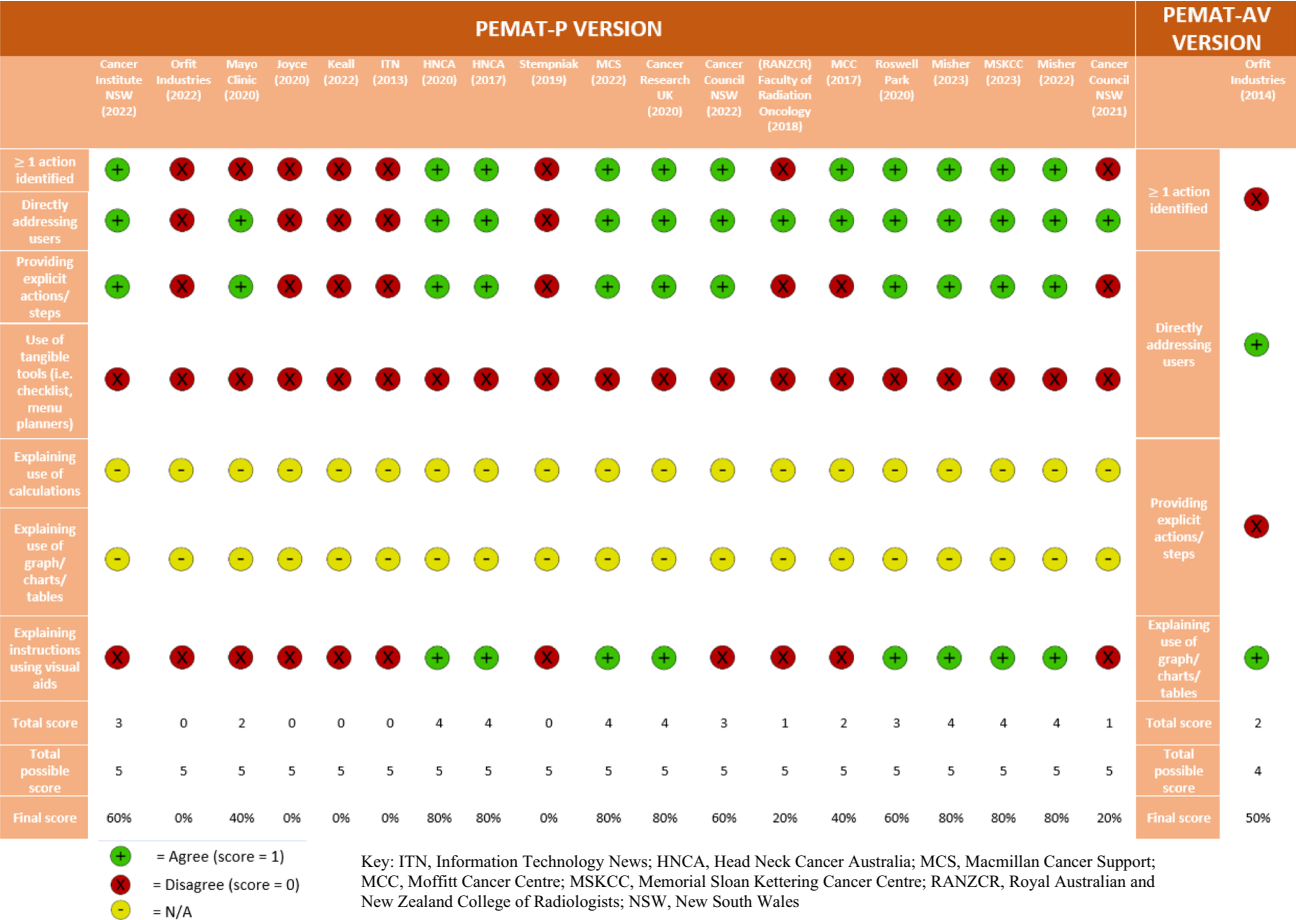
Understandability (blue) and actionability (orange) scores (PEMAT-P and PEMAT-AV VERSIONS) created based on Agency for Healthcare Research and Quality

All 19 printable PEMs assessed using the PEMAT-P scoring system were found to have used visual aids in their material, including a real-life demonstration, schematic or embedded audio-visual material (Fig. 2). No PEMs included calculations (Fig. 2). Similarly, no PEMs explained the use of charts, graphs, and diagrams to take action (Fig. 2).

Figure 2 presents the results for understandability and actionability of PEMs ordered by the Authoring body category. The median understandability score was 86% with minimum and maximum scores being 47% and 100% respectively. The Foundation/Organisation category (*n* = 10) had consistently higher scores for understandability and accounted for the upper half of the score distribution. All four PEMs achieving maximum understandability scores were produced by bodies within the Foundation/Organisation category. Government Body (*n* = 1) is also within the 50th percentile for understandability. The Other category (*n* = 6) consistently scored below the 25th percentile.

The median actionability score was 55% ranging between 0 and 80%. All six PEMs achieving maximum actionability scores were authored by the Foundation/Organisation. By contrast, all five PEMs obtaining minimum actionability scores belong to the Other category. Of the 20 PEMs included in this review, most generally scored higher for understandability compared to actionability.

**Discussion**

The review highlights key characteristics of PEMs available on the topic of IMs within Australia and internationally. Overall, there was wide variability between the PEMs, scoring from 0 to 80% for actionability and from 47 to 100% for understandability.

Of the 20 sources that fit the inclusion criteria, the highest-scoring PEMs were found from within Australia and the US and those that scored lowest were from Global sources. The purpose of IMs prevailed as the most included topic, followed by the mask-fitting process, with nearly all PEMs including a real-life demonstration of IMs, in video or image format. One-quarter of PEMs included audio-visual aids, which have been shown to improve patient comprehension, lower anxiety, and improve outcomes [37]. A total of four PEMs discussed open IMs. This could be argued as a weakness of the remaining 16 sources, as further education and the option of open-face masks could help to lower unwanted and negative patient experiences and mask anxiety [8]. However, RT providers may only use closed IMs and therefore do not provide information about open IMs. Sixteen (80%) of PEMs included links to other resources, which could be considered a strength, contributing to improving patient education. Less than half of the PEMs included information about preparation for the IM and radiation therapy. This may be seen as a gap in the included PEMs; however, physical preparation for head and neck RT is typically minimal. The focus here may be more specifically targeted towards mental preparedness, aiming to prevent anxiety around the mask-making process.

Head and Neck Cancer Australia and Misher (2022) each scored 100% for understandability and whilst nine other PEMs scored above 80%, Imaging Technology News (2013) and Orfit Industries (2022) scored lowest, with 47% and 56%, respectively [21–23, 29, 33]. Only six of the PEMs achieved 80% for actionability, and five scored 0%. One source was exclusively in audio-visual format but was unremarkable, scoring at 50% in both understandability and actionability in its specific PEMAT-AV scoring [32]. It was found that PEMs produced by Foundations/Organisations generally have higher understandability and actionability than other authoring bodies. By contrast, the Other category has poorer understandability and actionability scores than other authoring bodies.

Initial access to health care and treatment information may begin with patients reading online health information. To improve patient access to valuable PEMs, organisations and health systems should aim to provide highly understandable and actionable PEMs.

### Limitations

There are several limitations to this study. Geographic location, personalisation algorithms, and the continually updating nature of web browsers hinder the reproducibility of proposed search findings and can introduce bias [38, 39]. Lack of resources required searches to be limited to English language; as a result, a variety of PEMs in languages other than English may have been excluded. As a result, a vast amount of HNC education materials and patients remain unrepresented in this review. Regarding the comparison of understandability and actionability between different countries, small sample sizes from select countries may have affected conclusion validity. The readability of the included PEMs is another variable that was not considered in this review and would have aided in determining the overall quality of the PEMs included. The PEMAT tools used do not intend to assess the “accuracy, comprehensiveness, or cultural appropriateness” of the included PEMs [16]. These are important criteria to consider when assessing PEMs and their value to consumers; however, these variables are out of scope for this review.

The PEMAT tool utilised lists explicit scoring items; however, items scoring use of common language may be biased from the authors’ experience.

### Relevance of the Findings

Pre-treatment education is routine for HNC patients to ensure the patient has a thorough understanding of the treatment and accompanying processes, like IM making. This pre-treatment education alongside provided education materials can aid in obtaining informed consent from the patient [40]. The differences in IM-focused PEMs for HNC patients identified in this review suggest that some sources may be more thorough at educating patients about the RT procedure, side effects, and what the patient can expect, an important factor for both patients and treatment providers. Numerous studies discuss patient education as a tool to alleviate medical anxiety [40–45]. However, to the authors’ knowledge, no studies have investigated the effectiveness of PEMs in alleviating mask anxiety. Poor-quality PEMs, such as those with low understandability and actionability, may heighten a patient’s anxiety, placing more strain on healthcare providers. Quality PEMs are important to both patients and practitioners for this reason. PEMs included in this review tend to have higher understandability than actionability. This indicates that the actionability of most PEMs is subject to improvement. Authoring bodies such as Foundations and Treatment Providers focus more on incorporating coping mechanisms for patients to manage mask anxiety.

As the way health practitioners deliver educational material to patients changes and develops with technology, the industry must aim to include recent, valuable, and relevant information whilst developing and updating PEMs to improve the patient experience.

## Conclusion

Education materials available for HNC patients regarding IMs vary significantly in content, understandability, and actionability. Our study found that of the 20 materials analysed, each PEM addressed different aspects of RT and IMs. The materials were generally consistent in addressing the purpose of masks, providing real-life demonstrations of mask-wearing, and linking to other resources. It was commonly found that PEMs did not include information for patient preparation for RT or the IM procedure. Additionally, only a third of PEMs were found to have content on true patient experience.

Overall, providing effective PEMs disseminates accurate information and facilitates patients’ preparation for IM wearing. Current available PEMs on this topic vary in their quality and could be improved through assessment using tools such as the PEMAT-P. Further research may investigate how effective these available PEMs are in conveying information to patients and reducing mask anxiety.

## Data Availability

Data sharing is not applicable to this article because no datasets were generated or analysed for the review.
